# Modified method for differentiation of myeloid-derived suppressor cells *in vitro* enhances immunosuppressive ability via glutathione metabolism

**DOI:** 10.1016/j.bbrep.2022.101416

**Published:** 2022-12-28

**Authors:** Haoyang Zhou, Zhiqi Xie, Naosuke Morikawa, Fuminori Sakurai, Hiroyuki Mizuguchi, Daisuke Okuzaki, Naoki Okada, Masashi Tachibana

**Affiliations:** a Project for Vaccine and Immune Regulation, Graduate School of Pharmaceutical Sciences, Osaka University, Osaka, 565-0871, Japan; b Department of Pharmacology, School of Medicine, Zhejiang University City College, Zhejiang, 310015, China; c Laboratory of Biochemistry and Molecular Biology, Graduate School of Pharmaceutical Sciences, Osaka University, Osaka, 565-0871, Japan; d Laboratory of Hepatocyte Regulation, National Institutes of Biomedical Innovation, Health and Nutrition, Osaka, 567-0085, Japan; e Integrated Frontier Research for Medical Science Division, Institute for Open and Transdisciplinary Research Initiatives, Osaka University, 565-0871, Osaka, Japan; f Global Center for Medical Engineering and Informatics, Osaka University, Osaka, 565-0871, Japan; g Center for Infectious Disease Education and Research, Osaka University, Osaka, 65-0871, Japan; h Single Cell Genomics, Human Immunology, WPI Immunology Frontier Research Center, Osaka University, Osaka, 65-0871, Japan; i Genome Information Research Center, Research Institute for Microbial Diseases, Osaka University, Osaka, 565-0871, Japan; j Laboratory of Vaccine and Immune Regulation (BIKEN), Graduate School of Pharmaceutical Sciences, Osaka University, Osaka, 565-0871, Japan

**Keywords:** Myeloid-derived suppressor cell, Immunosuppression, Ly-6G, Tumor progression, BM-MDSC, Glutathione metabolism, Ab, antibody, BM, bone marrow, CTLA-4, cytotoxic T-lymphocyte-associated protein 4, FBS, fetal bovine serum, GM-CSF, granulocyte-macrophage colony-stimulating factor, ICI, immune checkpoint inhibitor, iNOS, inducible nitric oxide synthase, Ly-6G, lymphocyte antigen 6G, MDSCs, myeloid-derived suppressor cells, M-MDSCs, monocytic MDSCs, PBS, phosphate-buffered saline, PD-1, programmed cell death 1, PD-L1, programmed cell death 1 ligand 1, PMN-MDSCs, polymorphonuclear MDSCs, Cybb, Cytochrome *b*-245 beta polypeptide, Rb1, retinoblastoma 1, ROS, reactive oxygen species

## Abstract

Myeloid-derived suppressor cells (MDSCs), which accumulate in tumor bearers, are known to suppress anti-tumor immunity and thus promote tumor progression. MDSCs are considered a major cause of resistance against immune checkpoint inhibitors in patients with cancer. Therefore, MDSCs are potential targets in cancer immunotherapy. In this study, we modified an *in vitro* method of MDSC differentiation. Upon stimulating bone marrow (BM) cells with granulocyte-macrophage colony-stimulating factor *in vitro*, we obtained both lymphocyte antigen 6G positive (Ly-6G^+^) and negative (Ly-6G^−^) MDSCs (collectively, hereafter referred to as conventional MDSCs), which were non-immunosuppressive and immunosuppressive, respectively. We then found that MDSCs differentiated from Ly-6G^−^ BM (hereafter called 6G^−^ BM-MDSC) suppressed T-cell proliferation more strongly than conventional MDSCs, whereas the cells differentiated from Ly-6G^+^ BM (hereafter called 6G^+^ BM-MDSC) were non-immunosuppressive. In line with this, conventional MDSCs or 6G^−^ BM-MDSC, but not 6G^+^ BM-MDSC, promoted tumor progression in tumor-bearing mice. Moreover, we identified that activated glutathione metabolism was responsible for the enhanced immunosuppressive ability of 6G^−^ BM-MDSC. Finally, we showed that Ly-6G^+^ cells in 6G^−^ BM-MDSC, which exhibited weak immunosuppression, expressed higher levels of *Cybb* mRNA, an immunosuppressive gene of MDSCs, than 6G^+^ BM-MDSC. Together, these data suggest that the depletion of Ly-6G^+^ cells from the BM cells leads to differentiation of immunosuppressive Ly-6G^+^ MDSCs. In summary, we propose a better method for MDSC differentiation *in vitro*. Moreover, our findings contribute to the understanding of MDSC subpopulations and provide a basis for further research on MDSCs.

## Highlights

• We optimized a method for MDSC differentiation *in vitro*.
• Ly-6G^+^ cells differentiated from Ly-6G^−^ BM cells exhibited weak immunosuppression.
• Ly-6G^+^ cells differentiated from Ly-6G^−^ BM cells expressed high *Cybb* mRNA levels.
• Ly-6G^+^ depletion from BM cells led to immunosuppressive Ly-6G^+^ MDSC differentiation.
• Our findings improve our understanding of MDSC subpopulations for further research.

## Graphical abstract

**Figure:**
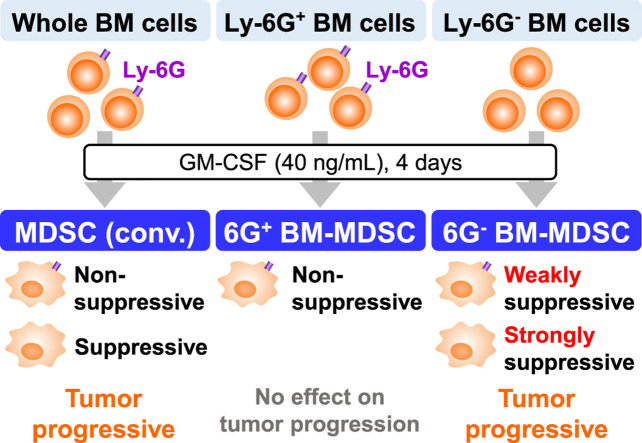

## 1 Introduction

Cancer is one of the major causes of death worldwide, which highlights the need to develop more effective cancer therapy. In the past decade, cancer immunotherapy that activates anti-cancer immune cells or inhibits the suppression of anti-cancer immune cells was developed. Such methods have exhibited prominent therapeutic effects with fewer side effects [1]. In particular, immune checkpoint inhibitors (ICIs) such as anti-programmed cell death 1 (PD-1) antibody (Ab) and anti-cytotoxic T-lymphocyte-associated protein 4 (CTLA-4) Ab have been reported for their revolutionary clinical outcomes [[2], [3], [4]]; they have become standard therapeutic agents for several types of cancer [5]. However, multiple studies have demonstrated that only a limited number of patients respond to anti-PD-1 or anti-CTLA-4 Abs [4,6]. Hence, to overcome this limitation, it is necessary to improve the therapeutic efficacy of ICIs.

Myeloid-derived suppressor cells (MDSCs) are an immature heterogeneous group of myeloid cells that expand in cancerous or inflammatory conditions and co-express CD11b and Gr-1 in mice with the ability to suppress T-cell proliferation [7,8]. MDSCs exacerbate tumors [9,10] and expand in patients with anti-PD-1 resistance or anti-CTLA-4 Abs [4,5,11]. This suggests that MDSCs are one of the causes of anti-PD-1 resistance or anti-CTLA-4 Abs [12]. Therefore, targeting MDSCs is a promising strategy for overcoming the limitations of ICIs.

Studies have reported that several inflammatory factors are involved in the differentiation, proliferation, and immunosuppressive function of MDSCs; for instance, cytokines such as granulocyte-macrophage colony-stimulating factor (GM-CSF) and granulocyte colony-stimulating factor (G-CSF) induced MDSC differentiation [7,13,14]. However, little is known about the MDSC differentiation pathway and its mechanism. MDSCs are classified into CD11b^+^Ly-6G^+^Ly-6C^int^ polymorphonuclear MDSCs (PMN-MDSCs) and CD11b^+^Ly-6G^−^Ly-6C^hi^ monocytic MDSCs (M-MDSCs), based on their morphology and expression of surface markers [7]. In addition, PMN-MDSCs suppress T-cell proliferation using arginase and ROS, while M-MDSCs do so using arginase and nitric oxide [15]. Besides, such differences in the immunosuppressive activity of these two subpopulations and their corresponding origins are less characterized. In tumor-bearing mice, PMN-MDSCs constitute the majority of circulating MDSCs [16,17], whereas M-MDSCs exhibit stronger immunosuppressive activity [10]. Targeting PMN-MDSC development, recruitment, or function improved the efficacy of immunotherapy [18], which also indicated PMN-MDSC immunosuppression. Therefore, differentiating PMN-MDSCs *in vitro* is important to the understanding of immunosuppression. However, only few studies have reported the immunosuppressive effects of PMN-MDSCs *in vitro*. Rößner et al. showed that only the Ly-6G^low^ cells enriched from MDSCs cultured *in vitro* were immunosuppressive, whereas the Ly-6G^high^ population was not [19]. Here, we investigated the differentiation and immunosuppressive ability of MDSC subpopulations *in vitro* and modified a differentiation method, which can potentially be used for further MDSC studies.

## 2 Materials and methods

### 2.1 Mice

All C57BL/6J female mice used for this study were 6–8 weeks of age at the start of the experiments and were purchased from Shimizu Laboratory Supplies (Kyoto, Japan). All mice were bred and maintained under specific pathogen-free conditions. All animal experimental procedures in this study were performed in accordance with the institutional guidelines for animal experiments at Osaka University, Japan (Douyaku R03-7-2).

### 2.2 Bone marrow (BM) mononuclear cell isolation

BM mononuclear cells were flushed out vigorously from the femur and tibia with 2% fetal bovine serum (FBS; Gibco, Waltham, MA, USA)/phosphate-buffered saline (PBS; Gibco). Cells were collected by centrifugation at 330×*g* for 5 min at 4 °C and then filtered through a 70-μm cell strainer. The erythrocytes were then removed by suspending in ammonium chloride-potassium (ACK) hemolytic buffer, followed by washing with 2% FBS/PBS.

### 2.3 Separation of Ly-6G^+^ and Ly-6G^−^ cells

The BM cells were blocked with TruStain fcX (anti-mouse CD16/32) antibody (Clone 93; BioLegend, San Diego, CA, USA) and stained with Biotin anti-mouse Ly-6G antibody (Clone 1A8; BioLegend) for 15 min at 4 °C. The cells were then washed and resuspended in 2% FBS/PBS and separated into Ly-6G^+^ and Ly-6G^−^ BM using a MojoSort magnetic cell separation system and MojoSort streptavidin nanobeads (BioLegend) following the manufacturer's instructions.

### 2.4 MDSC differentiation

BM cells obtained were suspended in RPMI-1640 (Sigma-Aldrich, Burlington, MA, USA) medium with GM-CSF (40 ng/mL; Peprotech, Cranbury, NJ, USA) supplemented with 10% FBS (Gibco), 2 mM GlutaMAX (Gibco), and 100 units/mL penicillin-streptomycin (Nacalai Tesque, Kyoto, Japan) at a concentration of 2.5 × 10^5^ cells/mL. Cell suspensions (10 mL) were then seeded in 100 mm tissue culture-treated dishes (Corning, Corning, NY, USA) and cultured at 37 °C in an atmosphere of 5% CO_2_ for 4 days. Later, the cells were harvested and used or separated into Ly-6G^+^ and Ly-6G^−^ cells and used for subsequent experiments.

### 2.5 Cell line

The EL4 lymphoma cell line was purchased from the American Type Culture Collection (ATCC, Manassas, VA, USA) and maintained in RPMI-1640 medium (FUJIFILM Wako, Tokyo, Japan) supplemented with 10% FBS and 100 units/mL penicillin-streptomycin. Cells were resuscitated and cultured following ATCC guidelines. The cells were used within one month of thawing from early passage (≤3 passages of the original vial) lots.

### 2.6 Murine tumor studies

The hair from the inoculation site of C57BL/6J mice was removed in advance. EL4 cells (2 × 10^5^ cells/mouse) were inoculated subcutaneously into the lower right flank of mice. Four days after the inoculation, MDSCs that differentiated *in vitro* were harvested, suspended in PBS, and injected once intravenously into EL4-bearing mice (3 × 10^6^ cells/mouse). The tumor volume and body weight of the mice were measured periodically; the volume was calculated using the following formula: tumor volume (cm^3^) = 0.523 × length (cm) × width (cm) ^2^.

### 2.7 T-cell suppression assay

The spleen harvested from the mice was ground to splenocytes and passed through a 70-μm cell strainer. The cells obtained were suspended in ACK hemolytic buffer to remove any erythrocytes and washed with 2% FBS/PBS to get spleen mononuclear cells. Following the protocol for MojoSort mouse CD4/CD8 nanobeads (BioLegend), these spleen mononuclear cells were then isolated as CD4^+^ and CD8^+^ T cells. The T cells were suspended in 0.2% BSA/PBS, an equal volume of 10 μM Cell Proliferation Dye eFlour670 (Thermo Fisher Scientific, Waltham, MA, USA) was added, and the cells were incubated at 37 °C for 10 min for staining. The cells were washed and suspended in RPMI-1640 medium supplemented with 10% FBS, 2 mM GlutaMAX, 100 units/mL penicillin-streptomycin, 10 mM HEPES (Gibco), MEM non-essential amino acids (FUJIFILM Wako), 1 mM sodium pyruvate (Gibco), and 55 μM 2-mercaptoethanol (Sigma-Aldrich). T cells were seeded in 96-well plates at a density of 1 × 10^5^ cells/200 μL per well. All wells were pre-coated with anti-CD3ϵ Ab (Clone 145-2C11; BioLegend), diluted with PBS to a concentration of 1 μg/mL, and stored at 4 °C overnight before use. MDSCs differentiated *in vitro* or separated were added to the wells at different T-cell ratios. For stimulation, anti-CD28 Ab (Clone 37.51; BioLegend) was also added to each well at a final concentration of 0.5 μg/mL. After 3 days of incubation at 37 °C in a 5% CO_2_ atmosphere, the proliferation of CD4^+^ and CD8^+^ T cells was analyzed using flow cytometry (BD FACSCanto II; BD Biosciences, San Jose, CA, USA).

### 2.8 Flow cytometry analysis

Cells were washed with 2% FBS/PBS, treated with TruStain FcX (anti-mouse CD16/32) antibody (Clone 93; BioLegend), and incubated at 4 °C for 5 min to block Fc receptors. Then, the cells were stained with fluorescently labeled antibodies (listed in Supplementary Table 1) at 4 °C under light-shielded conditions for 15 min. The cells were then washed and resuspended in 2% FBS/PBS containing 7-aminoactinomycin D as a viability stain (BioLegend) to remove dead cells during analysis. Flow cytometry analysis was performed using a BD FACSCanto II device (BD Biosciences), and the acquired data were analyzed using FlowJo software, version 10.6.2 (BD Biosciences).

### 2.9 Dendritic cell differentiation

BM cells were suspended in RPMI-1640 (Sigma-Aldrich) medium with GM-CSF (20 ng/mL; Peprotech) supplemented with 10% FBS (Gibco), 2 mM GlutaMAX (Gibco), and 100 units/mL penicillin-streptomycin (Nacalai Tesque) at a concentration of 2.5 × 10^5^ cells/mL. Cell suspensions (10 mL) were then seeded in 100 mm non-treated dishes (AGC TECHINO GLASS, Shizuoka, Japan) and cultured at 37 °C in an atmosphere of 5% CO_2_ for 8 days.

### 2.10 Microarray

Total RNA was extracted from cells using the miRNeasy Mini kit (Qiagen) according to the manufacturer's protocol. Total RNA (200 ng) was reverse-transcribed into double-stranded cDNA using AffinityScript multiple temperature reverse transcriptase (Agilent Technologies Inc., Palo Alto, CA). The resulting complimentary RNA (cRNA) was labeled with cyanine-3-labeled cytosine triphosphate (PerkinElmer, Wellesley, MA, USA) using a Low Input Quick-Amp Labeling kit (Agilent Technologies Inc.). One color experiment was performed by hybridizing four cRNAs onto an Agilent SurePrint G3 Mouse GE 8 × 60 K Microarrays (design ID 028005; Agilent Technologies Inc.). The Subio Platform and Subio Basic Plug-in (v1.12; Subio Inc., Aichi, Japan) were then used to calculate the between-sample fold change. Gene set enrichment analysis (GSEA) was used for the comparison of differentially enriched gene sets. The web tool “Calculate and draw custom Venn diagrams” [20] was used to create Venn diagrams.

### 2.11 Quantitative reverse transcription polymerase chain reaction (qRT-PCR)

Total RNA was extracted from MDSCs using ISOGEN (NIPPON GENE, Tokyo, Japan). After removing the genomic DNA with RNase-free DNase, a reverse transcription reaction was performed using the SuperScript VILO cDNA synthesis kit (Thermo Fisher Scientific). Using the synthesized cDNA as a template, PCR was performed using SYBR Premix Ex Taq II (Thermo Fisher Scientific) to amplify the cDNAs of various genes. The StepOnePlus Real-time PCR system (Thermo Fisher Scientific) was used for PCR and analysis. The primer sequences used are listed in Supplementary Table 2 mRNA expression was standardized using the mouse glyceraldehyde 3-phosphate dehydrogenase (*Gapdh*) gene and expression comparisons were performed using the *ΔΔCt* method.

### 2.12 Statistical analyses

Significant differences were assessed using Student's *t*-test or one- or two-way analysis of variance (ANOVA) using GraphPad Prism 7 (GraphPad Software, San Diego, CA, USA). *p* < 0.05 was considered to be statistically significant.

## 3 Results

### 3.1 Ly-6G^+^ subpopulation of MDSCs derived from BM cells exhibits no immunosuppression

**Fig. 1:**
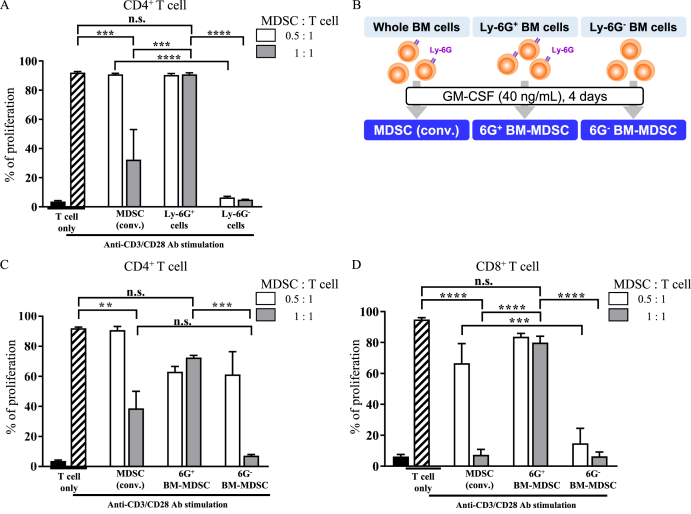
Conventional method cannot induce differentiation of immunosuppressive Ly-6G^+^ MDSCs (A) Different concentrations of MDSCs were co-cultured in a 1:1 or 0.5:1 ratio with eFluor 670-labeled CD4^+^ T cells, followed by stimulation using anti-CD3ε and anti-CD28 antibodies. Data represent means ± S.E.M. of three independent experiments (one-way ANOVA; n.s: not significant, ***p < 0.001 and ****p < 0.0001). (B) BM cells collected from C57BL/6J mice were cultured for 4 days upon stimulation with GM-CSF for the differentiation of MDSC (conv.), which includes Ly-6G^+^ cells. On the other hand, Ly-6G^+^ cell-selected or -depleted BM were cultured according to the protocol for *in vitro* MDSCs differentiation, hereinafter called 6G^+^ BM-MDSCs and 6G^−^ BM-MDSCs, which includes Ly-6G^+^ cells. Different concentrations of MDSCs were combined with eFluor 670-labeled CD4^+^ (C) or CD8^+^ (D) T cells, followed by stimulation with anti-CD3ε and anti-CD28 antibodies. Data represent mean ± S.E.M. of three independent experiments (one-way ANOVA; n. s.: not significant, **p < 0.01, ***p < 0.001, and ****p < 0.0001).

Previous studies have developed a method that differentiates BM cells into MDSCs *in vitro* upon GM-CSF stimulation (hereafter referred to as *in vitro* MDSCs) [21,22]. Following this method, we investigated the immunosuppressive ability of CD11b^+^Ly-6G^+^ MDSCs and CD11b^+^Ly-6G^−^ MDSCs. These MDSCs were enriched based on Ly-6G expression and co-cultured with CD4^+^ T cells. CD11b^+^Ly-6G^+^Ly-6C^int^ cells (hereafter referred to as Ly-6G^+^ cells) enriched from *in vitro* MDSCs showed negligible immunosuppression, indicating that these cells were not immunosuppressive. In contrast, CD11b^+^Ly-6G^−^ cells (hereafter referred to as Ly-6G^−^ cells) showed stronger immunosuppression than unseparated MDSCs (Fig. 1A). Since Ly-6G^+^ cells were not immunosuppressive, we investigated whether their depletion from BM cells at the beginning of *in vitro* MDSC differentiation resulted in the differentiation of Ly-6G^+^ MDSCs. Ly-6G^+^ and Ly-6G^−^ BM cells were enriched and cultured for 4 days upon GM-CSF stimulation (Fig. 1B), and the immunosuppressive activity of each group of MDSCs was investigated by co-culture with T cells. The proliferation of both CD4^+^ and CD8^+^ T cells was not suppressed by MDSCs derived from Ly-6G^+^ BM cells, hereinafter referred to as 6G^+^ BM-MDSC, suggesting that Ly-6G^+^ BM cells could not acquire immunosuppressive ability during 4-day cultivation (Fig. 1C and D). In contrast, *in vitro* MDSCs differentiated from unseparated BM cells (hereafter referred to as MDSCs (conv.)) and MDSCs cultured from Ly-6G^−^ BM cells (hereafter referred to as 6G^−^ BM-MDSC) showed immunosuppressive activity (Fig. 1C and D). In addition, 6G^−^ BM-MDSC were able to suppress CD8^+^ T cells more strongly at a ratio of 0.5:1 than MDSCs (conv.) (Fig. 1D). Taken together, our results show that *in vitro* MDSCs with stronger immunosuppressive activity can be obtained by differentiating MDSCs from Ly-6G^−^ BM cells.

### 3.2 6G^+^ BM-MDSC exhibit no ability to promote tumor progression

**Fig. 2:**
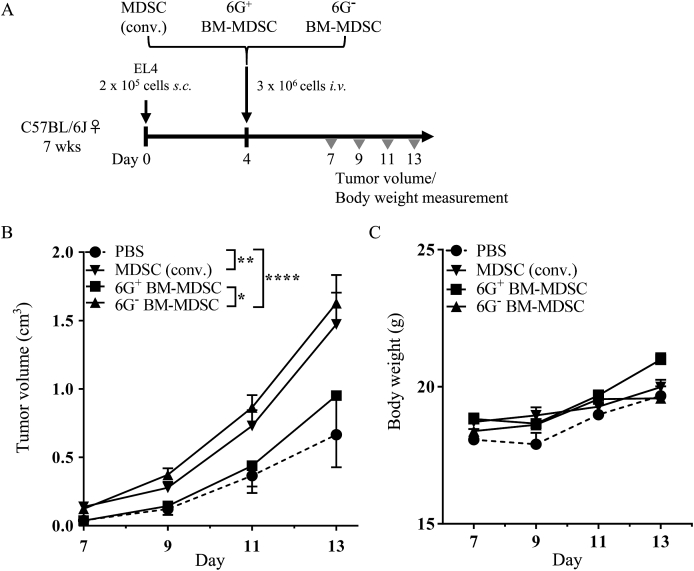
6G^+^ BM-MDSCs exhibit impaired ability to promote tumor progression (A) Four days after inoculation of EL4 cells, mice received PBS (n = 6), MDSCs (conv., n = 5), 6G^+^ BM-MDSCs (n = 3), or 6G^−^ BM-MDSCs (n = 7). (B) Tumor volume and (C) body weight were measured. Data are means ± S.E.M. (two-way ANOVA; **p < 0.01 and ***p < 0.001).

Previous studies have shown that MDSC transfer promotes tumor progression *in vivo* [23,24]. To evaluate the immunosuppressive ability *in vivo*, MDSCs cultured *in vitro* using each differentiation method were intravenously transferred into EL4 tumor-bearing mice (Fig. 2A). Compared to that in the PBS control group, tumor progression was promoted in MDSC (conv.)-transferred mice and 6G^−^ BM-MDSC-transferred mice. Notably, the transfer of 6G^+^ BM-MDSC did not affect tumor progression (Fig. 2B), further supporting that 6G^+^ BM-MDSC are not immunosuppressive. However, the body weight of the mice did not change post MDSC transfer, indicating the absence of toxic reactions (Fig. 2C). Together, these data suggest that, consistent with our findings *in vitro*, 6G^+^ BM-MDSC (but not MDSCs (conv.) and 6G^−^ BM-MDSC) showed no immunosuppressive ability *in vivo* also and failed to acquire immunosuppressive ability even in tumor-bearing mice.

### 3.3 6G^−^ BM-MDSC exhibit stronger immunosuppression

**Fig. 3:**
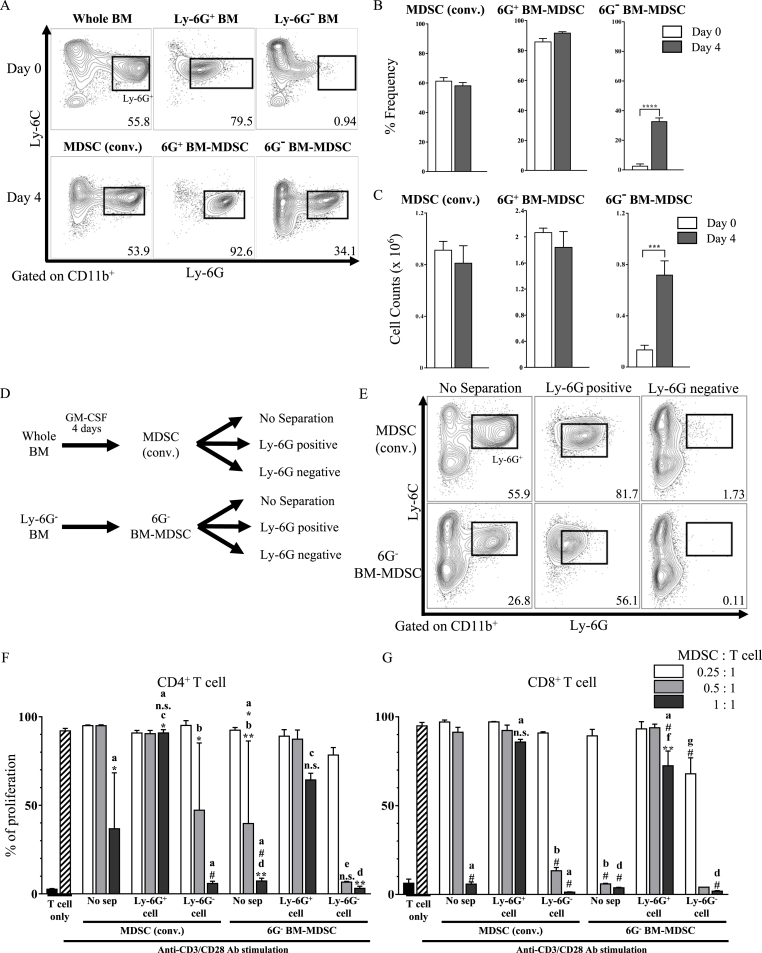
*In vitro* differentiation in the absence of Ly-6G^+^ BM cells results in MDSCs with stronger immunosuppressive abilities (A) Flow cytometric analysis of the frequencies of Ly-6G^+^ population among CD11b^+^ cells. Data represent four independent experiments. Percentage (B) and cell counts (C) of live Ly-6G^+^ population. Data are shown as mean ± S.E.M. values over four experiments. (Student's *t*-test: ***p < 0.001 and ****p < 0.0001). (D) Ly-6G^+^ or Ly-6G^**−**^ cells were collected from MDSC (conv.) or 6G^**−**^ BM-MDSC and were cultured according to the protocol for *in vitro* MDSC differentiation. (E) Flow cytometric analysis of the frequency of PMN-MDSCs and M-MDSCs among CD11b^+^ cells. Data represent two independent experiments. Different concentrations of MDSCs were combined in a 1:1, 0.5:1, or 0.25:1 ratio with eFluor 670-labeled CD4^+^ (F) or CD8^+^ (G) T cells, followed by stimulation with anti-CD3e and anti-CD28 Abs. Data represent means ± S.D. of two independent experiments (one-way ANOVA; ‘a’ vs T cell only; ‘b’ vs MDSC (conv.)-No sep (0.5:1); ‘c’ vs MDSC (conv.)-No sep (1:1); ‘d’ vs Ly-6G^+^ cell (1:1) in 6G^**−**^ BM-MDSC; ‘e’ vs 6G^**−**^ BM-MDSC-No sep (0.5:1), ‘f’ vs Ly-6G^+^ cell (1:1) in MDSC (conv.)'g’ vs Ly-6G^+^ cell (0.25:1) in MDSC (conv.); n. s, not significant; *p < 0.05, **p < 0.01, and ^#^*p* < 0.0001). No Sep, No separation.

The results thus far suggest that detailed analyses of MDSCs would improve the *in vitro* differentiation method. Hence, we looked at the subpopulations of MDSCs (conv.), 6G^+^ BM-MDSC, and 6G^−^ BM-MDSC. The proportion (Fig. 3A and B) and number (Fig. 3C) of the Ly-6G^+^ population in both MDSC (conv.) and 6G^+^ BM-MDSC did not significantly change during the 4-day cultivation period. In contrast, in the 6G^−^ BM-MDSC, more than 30% of cultured Ly-6G^−^ BM cells acquired the expression of Ly-6G (Fig. 3A–C), suggesting that Ly-6G^−^ BM cells partially differentiated into Ly-6G^+^ cells. To investigate the immunosuppressive function of each subpopulation of *in vitro* MDSCs, Ly-6G^+^ or Ly-6G^**−**^ cells were enriched from MDSC (conv.) or 6G^**−**^ BM-MDSC (Fig. 3D). Although Ly-6G levels were lower in Ly-6G^+^ cells because of the same clone of anti-Ly-6G used for purity check, Ly-6G^+^ or Ly-6G^**−**^ cells were successfully enriched in MDSCs (Fig. 3E). The enriched Ly-6G^+^ or Ly-6G^−^ cells were co-cultured with T cells. At the ratio of MDSCs to T cells (0.5:1), the 6G^−^ BM-MDSC without separation more strongly suppressed T-cell proliferation than MDSC (conv.) without separation (Fig. 3F and G). Ly-6G^+^ cells in 6G^−^ BM-MDSC showed no immunosuppression in CD4^+^ T cells but tended to be more strongly suppressed than 6G^+^ cells in MDSC (conv.) (Fig. 3F). In addition, at 1:1 ratio of MDSCs to T cells, Ly-6G^+^ cells in MDSC (conv.) did not show the ability to suppress T-cell proliferation, whereas Ly-6G^+^ cells in 6G^−^ BM-MDSC showed relatively weak immunosuppression in CD8^+^ T cells (Fig. 3G). These observations suggest that 6G^−^ BM cells partially differentiated into PMN-MDSCs. Ly-6G^−^ cells in both MDSC (conv.) and 6G^−^ BM-MDSC showed strong immunosuppression. Together, these data suggest that MDSCs with stronger immunosuppressive abilities can be differentiated *in vitro* in the absence of Ly-6G^+^ BM cells.

### 3.4 Immunosuppressive activity of MDSCs may require ROS production and down-regulation of retinoblastoma 1 (*Rb1*)

**Fig. 4:**
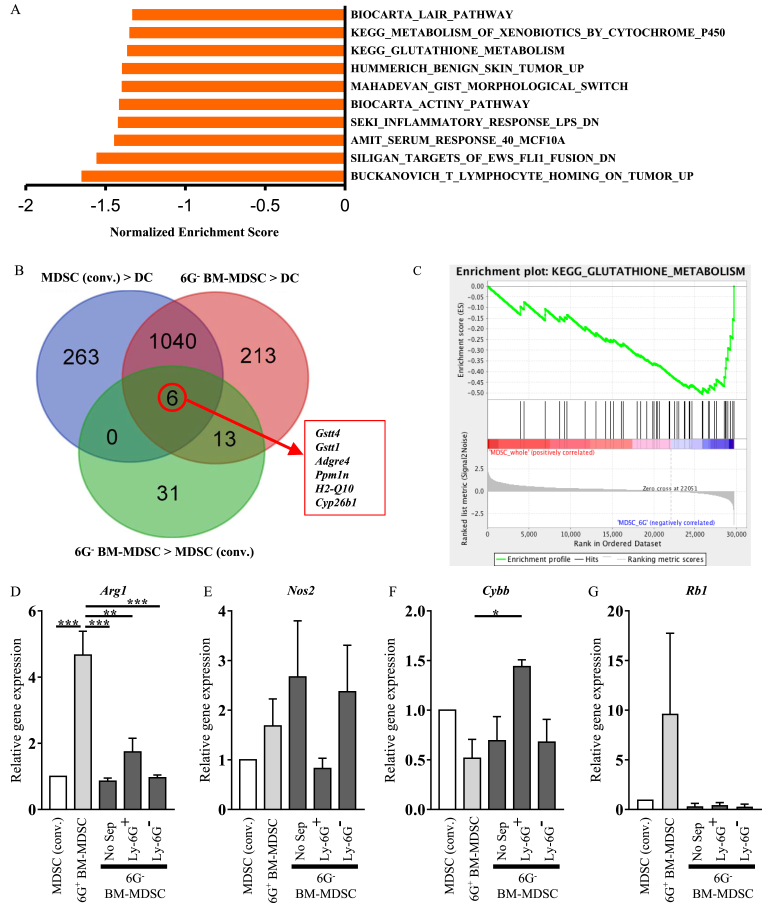
ROS production and *Rb1* down-regulation contribute to immunosuppressive ability of *in vitro* MDSCs Total RNA was extracted from MDSC (conv.), 6G^−^ BM-MDSC, and DCs, and microarray was performed. (A) Gene set enrichment analysis (GSEA) of differentially enriched gene sets in 6G^−^ BM-MDSC compared to MDSC (conv.). Top 10 gene sets are shown. (B) Venn diagram summarizing the overlap between genes highly expressed in MDSC (conv.) compared to DCs (blue circle), in 6G^−^ BM-MDSC compared to DCs (red circle), and in 6G^−^ BM-MDSC compared to MDSC (conv.) (green circle). (C) GSEA enrichment plot of glutathione metabolism by the comparison between MDSC (conv.) and 6G^−^ BM-MDSC. Total RNA was extracted from MDSCs *in vitro* and qRT-PCR analysis was performed to measure *Arg1* (D)*, Nos2* (E)*, Cybb* (F)*,* and *Rb1* (G) mRNA expression. Data are presented as mean ± S.E.M. of three independent experiments (one-way ANOVA; *p < 0.05, **p < 0.01, and ***p < 0.001). No Sep, No separation. . (For interpretation of the references to color in this figure legend, the reader is referred to the Web version of this article.)

To investigate the underlying mechanisms of the immunosuppressive ability of 6G^−^ BM-MDSC, we performed microarray followed by GSEA. Comparing the gene expression profiles of MDSC (conv.) and 6G^−^ BM-MDSC, we observed that several gene sets, such as those associated with inflammation and metabolism, were enriched in 6G^−^ BM-MDSC (Fig. 4A). Next, to identify the genes responsible for the enhancement of the immunosuppressive activity of 6G^−^ BM-MDSC, we compared these MDSCs with BM-derived dendritic cells (DCs), which are differentiated upon stimulation of GM-CSF for 8 days. We identified 1046 genes that were highly expressed in MDSC (conv.) and 6G^−^ BM-MDSC compared to DCs. Moreover, six genes were highly expressed in 6G^−^ BM-MDSC, and among these genes, glutathione S-transferase, that is, glutathione S-transferase θ 1 (*Gstt1*) and glutathione S-transferase θ 4 (*Gstt4*) were supposed to be the genes responsible for the enhancement of the immunosuppressive activity of 6G^−^ BM-MDSC (Fig. 4B). Then, GSEA further confirmed that the glutathione metabolism gene set was enriched in 6G^−^ BM-MDSC (Fig. 4A, C). Since glutathione S-transferase degrades glutathione, which is a ROS scavenger, these results suggest that ROS are critical for the enhanced immunosuppressive ability of 6G^−^ BM-MDSC.

Since the bulk microarray analysis did not determine the differentially expressed immunosuppressive molecules, we measured their mRNA expression in each MDSC subpopulation. Compared to MDSC (conv.), 6G^−^ BM-MDSC, and Ly-6G^−^/Ly-6G^+^ cells separated from 6G^−^ BM-MDSC, the expression of arginase 1 (*Arg1*) was higher in 6G^+^ BM-MDSC (Fig. 4D). There was no significant difference in the expression of inducible nitric oxide synthase (*Nos2*) in each MDSCs. However, the expression of *Nos2* tended to be higher in Ly-6G^−^ cells in 6G^−^ BM-MDSC but lower in Ly-6G^+^ cells in 6G^−^ BM-MDSC than in the others (Fig. 4E). Notably, the expression of NADPH oxidase 2 (*Cybb*), a ROS-producing enzyme, was significantly higher (*p* = 0.0315) in Ly-6G^+^ cells in 6G^−^ BM-MDSC compared to 6G^+^ BM-MDSC (Fig. 4F). In addition, the expression of *Rb1* tended to be down-regulated in Ly-6G^+^ cells in 6G^−^ BM-MDSC compared to that in 6G^+^ BM-MDSC (Fig. 4G). *Rb1* is a tumor suppressor gene [25]; the downregulation of *Rb1* expression leads to the differentiation of PMN-MDSCs from M-MDSCs [26]. Therefore, these results suggest that the immunosuppressive ability of Ly-6G^+^ cells in 6G^−^ BM-MDSC requires upregulation of *Cybb* and possibly downregulation of *Rb1*. Taken together, our results suggest that high levels of glutathione S-transferase and *Cybb* expression would promote ROS production, resulting in the enhanced immunosuppressive ability of 6G^−^ BM-MDSC.

## 4 Discussion

Our study shows that Ly-6G^+^ cells in MDSC (conv.) phenotypically resemble PMN-MDSCs but are not immunosuppressive. Zhu et al. had earlier found that *ex vivo*-induced PMN-MDSCs fail to suppress T cells [27]. On the other hand, it is also known that PMN-MDSCs from tumor-bearing mice, which despite being less immunosuppressive than M-MDSCs, are clearly defined as immunosuppressive [16,28]. Here, using our newly developed *in vitro* MDSC differentiation method and cultivation of Ly-6G^−^ BM cells, we obtained immunosuppressive Ly-6G^+^ cells, that is, PMN-MDSCs. Although these Ly-6G^+^ cells differentiate from Ly-6G^−^ BM cells, the presence of non-immunosuppressive Ly-6G^+^ cells, possibly neutrophils, inhibits their differentiation. Therefore, the removal of Ly-6G^+^ cells before *in vitro* MDSC differentiation should be considered. This point might be supported by Haverkamp et al. who reported that M-MDSCs are the dominant suppressive population and that removal of the neutrophilic lineage might be important to differentiate MDSCs [29]. However, the mechanisms by which neutrophils inhibit the differentiation of PMN-MDSCs remain to be addressed. Rößner et al. found that only the Ly-6G^low^ cells enriched from MDSCs cultured *in vitro* were immunosuppressive, but the Ly-6G^high^ population was not [19]. All these findings indicate that the conventionally differentiated Ly-6G^+^ cells that phenotypically correspond to PMN-MDSCs are not immunosuppressive, unlike PMN-MDSCs obtained from tumor-bearing hosts. Our findings complement the above idea and improve the current understanding of *in vitro* differentiation of PMN-MDSCs. However, it is still not known whether the tumor microenvironment plays a role in MDSC differentiation. Thus, the role of the tumor microenvironment in the early stages of *in vitro* MDSC differentiation requires further elucidation.

In the present study, correlating with their strong immunosuppressive ability, 6G^−^ BM-MDSC and its Ly-6G^−^ cells tended to show higher *Nos2* expression. These results are consistent with previous findings that *Nos2* plays an important role in the immunosuppressive ability of M-MDSCs with the Ly-6G^−^ phenotype [15]. Moreover, we found upregulated *Cybb* expression in Ly-6G^+^ cells in 6G^−^ BM-MDSC compared to that in 6G^+^ BM-MDSC, which might contribute to their immunosuppressive activity. Interestingly, the loss of RB1 protein in MDSCs is related to PMN-MDSC differentiation from M-MDSCs [26]. In our study, *Rb1* expression was low in Ly-6G^+^ cells in 6G^−^ BM-MDSC, which differed from that of 6G^+^ BM-MDSC. In conclusion, the downregulation of *Rb1* expression might be a potential indicator of *in vitro* differentiation of immunosuppressive PMN-MDSCs. However, further studies are needed to define the immunosuppressive mechanisms of these Ly-6G^+^ cells.

The analysis of gene expression profiles showed that glutathione metabolism was the pathway responsible for the enhanced immunosuppressive ability of 6G^−^ BM-MDSC. We previously showed that glutamate, one of the metabolites of glutathione, would enhance the immunosuppressive function of MDSCs [14]. In addition, we found that glutamate signaling through metabotropic glutamate receptor 2/3 enhances the immunosuppressive ability of MDSCs [30]. Altogether, these results suggested that glutamate would be an important factor in enhancing the immunosuppressive ability of 6G^−^ BM-MDSC.

Our study also suggested that the stimulation of BM cells by GM-CSF in the absence of Ly-6G^+^ cells could lead to the differentiation of stronger immunosuppressive MDSCs. Kumar et al. summarized the therapeutic effects of GM-CSF in cancer immunotherapy [31]. They also mentioned the pathogenic effects, including reprograming macrophages to the tumor-promoting M2 phenotype and the induction of MDSC differentiation. Most importantly, they listed some clinical cases that used GM-CSF to rescue tumor therapy-induced neutropenia. We previously reported that G-CSF, which is used for the prevention and therapy for neutropenia, enhanced the immunosuppressive activity of MDSCs through the glutathione degradation pathway, leading to tumor progression [14]; therefore, GM-CSF might also do so. Although further *in vivo* cancer model studies are needed, our findings indicated that such GM-CSF treatment might lead to the differentiation of stronger immunosuppressive MDSCs. Therefore, our results contribute to the investigation of the immuno-toxic effect of GM-CSF treatment in tumor therapy.

To gain a more comprehensive and detailed understanding of the characteristics and functions of MDSCs, a method for differentiating MDSCs *in vitro* that resembles those *in vivo* is essential. Our findings suggest an *in vitro* method for differentiating PMN-MDSCs and more immunosuppressive M-MDSCs in the absence of Ly-6G^+^ BM cells. To summarize, we provide a potential *in vitro* method to study *in vivo* PMN-MDSCs, which would be necessary for developing strategies in the future for accurately studying the MDSC subpopulations. We believe that our method would be helpful to overcome the limitations of ICIs and contribute to the understanding of cancer immunity.

## Funding

This work was supported in part by 10.13039/501100001691JSPS KAKENHI Grant Numbers JP19H04049 and JP22H03533 (Grants-in-aid for Scientific Research (B)) (M.T.). This research was also partially supported by the 10.13039/100009619Platform Project for Supporting Drug Discovery and Life Science Research (Basis for Supporting Innovative Drug Discovery and Life Science Research (BINDS)) from AMED under grant numbers JP22am121052 and JP22am121054.

## Declaration of competing interest

The authors declare that they have no known competing financial interests or personal relationships that could have appeared to influence the work reported in this paper.
