# Supplementary material for: Modified method for differentiation of myeloid-derived suppressor cells in vitro enhances immunosuppressive ability via glutathione metabolism
Source: Biochem Biophys Rep. 2022 Dec 28;33:101416. doi: 10.1016/j.bbrep.2022.101416 (PMC9807831; doi:10.1016/j.bbrep.2022.101416)
Supplement: Multimedia component 1 [file mmc1.docx]

**Supplementary Table 1.** List of fluorescently labeled antibodies used in flow cytometry cell staining

| Markers/Label | Clone | Manufacture |
| --- | --- | --- |
| Anti-mouse Ly-6G/Ly-6C (Gr-1)/BV510  Anti-mouse CD11b/APC  Anti-mouse CD45/PB  Anti-mouse Ly-6C/APC-Cy7  Anti-mouse Ly-6G/FITC  Anti-mouse CD4/PB  Anti-mouse CD8α/FITC | RB6-8C5  M1/70  30-F11  HK1.4  1A8  RM4-4  53-6.7 | BioLegend  BioLegend  BioLegend  BioLegend  BioLegend  BioLegend  BioLegend |

APC, Allophycocyanin; PB, Pacific Blue; BV510, Brilliant Violet 510; APC-Cy7, Allophycocyanin-Cyanine7; FITC, Fluorescein isothiocyanate

**Supplementary Table 2.** List of primer sequences used in qRT-PCR analysis

| **Gene** | **Forward (5ʹ to 3ʹ)** | **Reverse (5ʹ to 3ʹ)** |
| --- | --- | --- |
| *Gapdh* | TGACCTCAACTACATGGTCTACA | CCGTGAGTGGAGTCATACTGG |
| *Arg1* | CCTATGTGTCATTTGGGTGGATG | GGTTGTCAGGGGAGTGTTGAT |
| *Nos2* | GGAGTGACGGCAAACATGACT | TAGCCAGCGTACCGGATGA |
| *Cybb* | CCTCTACCAAAACCATTCGGAG | CTGTCCACGTACCGGATGA |
| *Rb1* | CAGGGCTGTGTTGACATCGGAGTA | TCCACGGGAAGGACAAATCTGTTC |

qRT-PCR, quantitative reverse transcription-polymerase chain reaction; Genes: *Gapdh*, glyceraldehyde 3-phosphate dehydrogenase; *Arg1*, arginase 1; *Nos2*, nitic oxide synthase 2; *Cybb*, cytochrome b-245 beta polypeptide; *Rb1*, retinoblastoma 1
